# Gluteal muscle composition differentiates fallers from non-fallers in community dwelling older adults

**DOI:** 10.1186/1471-2318-14-37

**Published:** 2014-03-25

**Authors:** Mario Inacio, Alice S Ryan, Woei-Nan Bair, Michelle Prettyman, Brock A Beamer, Mark W Rogers

**Affiliations:** 1 Department of Physical Therapy and Rehabilitation Science, University of Maryland School of Medicine, Baltimore, MD, USA; 2 Division of Gerontology and Geriatric Medicine, Baltimore VAMC GRECC and Department of Medicine, University of Maryland School of Medicine, Baltimore, MD, USA

**Keywords:** Older age, Falls, Muscle composition, Muscle performance

## Abstract

**Background:**

Impaired balance, loss of mobility and falls are major problems associated with changes in muscle in older adults. However, the extent to which muscle composition and related performance measures for different lower limb muscles are associated with falls in older individuals is unclear. This study evaluated lower limb muscle attenuation, intramuscular adipose tissue (IMAT) infiltration and muscle performance in older fallers and non-fallers.

**Methods:**

For this cross-sectional study, fifty-eight community dwelling older individuals (>65 years) were classified into fallers (n = 15) or non-fallers (n = 43). Computed tomography (CT) was used to determine muscle attenuation and intramuscular adipose tissue (IMAT) of multiple thigh and hip muscles. Muscle performance was assessed with isokinetic dynamometry.

**Results:**

For both groups, Rectus Femoris showed the highest muscle attenuation and lowest IMAT infiltration, and Gluteus Maximus and Gluteus Medius/Minimus muscles had the lowest muscle attenuation and highest IMAT infiltration. Fallers exhibited lower muscle attenuation and higher IMAT infiltration than non-faller participants in most muscles, where the gluteal muscles were the most affected (*p* < 0.05). Fallers also showed a lower peak hip abduction torque (*p* < 0.05). There were significant associations (*r* = 0.31 to 0.53) between joint torques and muscle composition, with the strongest associations between Gluteus Medius/Minimus and hip abduction strength.

**Conclusions:**

While fallers were generally differentiated from non-fallers by muscle composition, the most affected muscles were the proximal gluteal muscles of the hip joint accompanied by lower hip abduction strength, which may contribute to impaired balance function and increased risk for falls.

## Background

Among older adults, falls and related injuries are major health care problems that diminish quality of living and contribute to increased morbidity and mortality [1-4]. Thus, identifying the characteristics of individuals at higher risk for falls in order to establish effective falls prevention interventions is of major clinical significance [5-8].

Impairments of balance and mobility are important risk factors contributing to age-associated falls [8,9]. During everyday activities, protective stepping is normally a commonly used strategy for stabilizing balance in response to postural challenges. However, balance control through stepping is problematic for many older individuals, especially in the mediolateral (M-L) direction where vulnerability to imbalance and fall-related injuries may be heightened [8,10]. For example, when steps were randomly evoked in 12 different directions by waist-pull postural perturbations, an age-associated reduction in balance recovery effectiveness through stepping was identified particularly for the lateral direction among prospectively identified fallers [11]. A possible reason for diminished lateral balance function may be that older individuals less often engage in activities, such as single limb weight bearing tasks, sideways whole-body movements and other tasks that require vigorous use of the hip abductor-adductor muscles important for lateral balance control [10,12-14]. In this regard, older individuals have lower maximum isokinetic hip abduction torque implicated in lateral balance control than younger adults, and these age-related decrements in strength are greater for older individuals at higher risk for falls than for those at lower fall risk [7,15]. Although arthritic degeneration with older age can affect muscle composition [16], it is conceivable that the age-related reductions in physical activity patterns may also impact changes in muscle composition that affect balance and mobility particularly involving the mediolateral direction.

Aging brings about changes in skeletal muscle frequently manifested as primary sarcopenia [17]. Primary sarcopenia is characterized by an age-related reduction in muscle mass, strength, quality [18-20] and altered metabolism [21]. These changes often include increased intramuscular adipose tissue (adipose tissue within the epimysium (IMAT)) [22-24] and are associated with physical frailty, loss of mobility and increased risk for fractures [25,26]. Furthermore, IMAT infiltration and the density of the skeletal muscle fibers (muscle attenuation) may differentially affect the lower limb musculature [16,21,27].

Although sarcopenia and its associated muscle composition characteristics have received much attention, further clarification is needed concerning their relationship to clinically relevant functional impairments such as diminished muscular performance, balance function and the propensity to fall. Hence, the purpose of this study was to evaluate muscle attenuation and infiltration of IMAT by computed tomography (CT), and muscular performance in older fallers and non-fallers. We hypothesized that the proximal muscles of the hip would demonstrate greatest age-related changes in muscle composition. Furthermore, we anticipated that worse muscle composition would be associated with poorer muscular performance and that elderly fallers would show greater impairments in muscle composition and muscular performance than non-fallers.

## Methods

### Subjects

Participants were recruited mainly by community older adult newspaper advertisements in the Baltimore/Washington area. In order to recruit a sample of otherwise healthy community dwelling older individuals that would be able to undergo muscle composition and strength testing procedures without major confounding factors, participants aged 65 years or older were screened over the telephone by the recruitment staff, followed by a medical examination performed by a physician geriatrician. Exclusion criteria consisted of the following: 1) cognitive impairment (Folstein Mini Mental Score Exam < 24); 2) sedative use, such as Valium, Xanax or Lunesta; 3) unable to walk independently without the use of an assistive device; 4) any clinically significant functional impairment related to musculoskeletal, neurological, cardiopulmonary, metabolic, or other general medical problem; 5) engaged in a dedicated and structured physical exercise regimen for 3 or more days per week beyond more routine casual or recreational physical activity; and 6) Centers for Epidemiological Studies Depression Survey score greater than 16. The falls assessment was performed during the telephone screen and consisted of the fall history in the 12 months prior to enrollment. A fall was identified based on the criteria adopted by the World Health Organization [28]. This assessment identified 43 individuals (n = 21 males, n = 22 females) to be non-fallers and 15 (n = 5 males, n = 10 females) to be fallers. Descriptive information about the study participants is presented in Table 1. All subjects provided written informed consent that was approved by the research ethics committee from the Institutional Review Board of University of Maryland, Baltimore and the Baltimore Veteran’s Administration Research and Development prior to participation (HP-00040282).

**Table 1:** **Comparison between non**-**faller and faller groups on the sample demographics**

	**Non**-**fallers**	**Fallers**	***p***
	**( *n *** **= *43 *)**	**( *n *** **= *15 *)**	
Age (yr)	74.0 ± 1.1	73.0 ± 1.1	*0.56*
Height (*cm*)	166.9 ± 1.3	165.6 ± 2.5	*0.63*
Weight (*kg*)	75.1 ± 2.2	79.0 ± 5.0	*0.42*
*BMI* (*kg.m*^−*2*^)	26.7 ± 0.5	28.2 ± 1.3	*0.21*
Fat Mass (*kg*)	26.7 ± 1.1	31.0 ± 2.5	*0.09*
Fat-Free Mass (*kg*)	45.8 ± 1.4	47.2 ± 3.4	*0.65*
% Fat Mass	35.4 ± 0.9	38.3 ± 1.7	*0.12*
**Co-morbidities (%)**			
Hypertension	22.4	13.8	*0.27*
Hyperlipidemia	31.0	19.0	*0.19*
Diabetes	3.4	1.7	*0.56*
Coronary disease	1.7	3.4	*0.56*
Edema (lower limbs)	3.4	3.4	*1.00*
Painful joints	19.0	15.5	*0.65*
Osteoarthritis	24.1	13.8	*0.20*
Osteoporosis	10.3	6.9	*0.53*
Depression	1.7	6.9	*0.18*
Incontinence	1.7	1.7	*1.00*
Asthma	5.2	1.7	*0.32*
**Number of reported falls in previous 12 months (%)**			
1	0	66.7	
2	0	26.7	
3	0	6.7	

Data is expressed as Mean ± SEM. Comparisons significant for *p* < 0.05.

### Procedures

**Figure 1:**
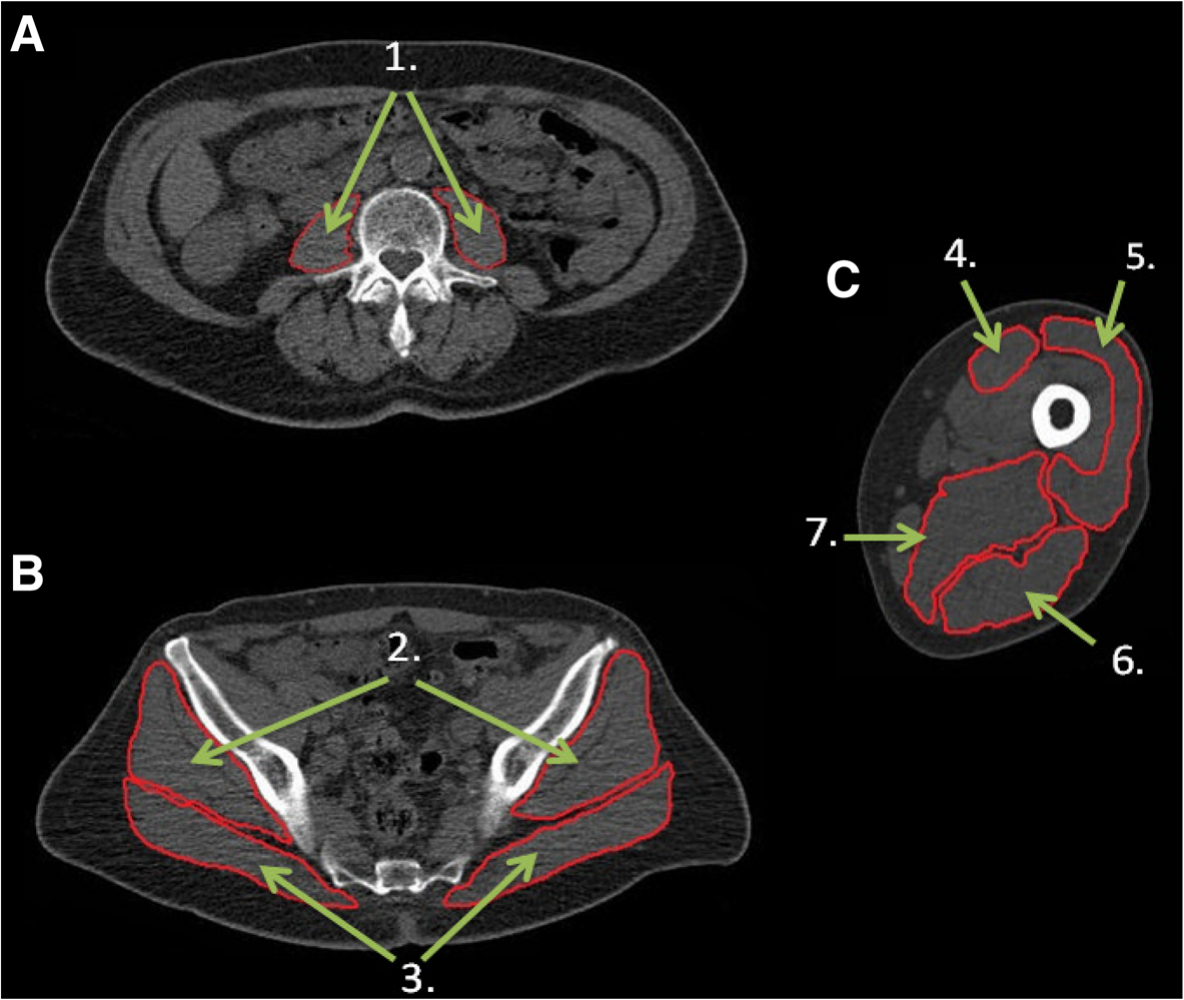
**Representative example of computed tomography (CT) scans from an older adult showing A) abdominal scan; B) hip scan; C) thigh scan.** 1. Psoas; 2. Gluteus Medius and Minimus; 3. Gluteus Maximus; 4. Rectus Femoris; 5. Vastus Lateralis; 6. Hamstrings compartment; 7. Adductor Magnus and Longus.

This study adopted a single session cross-sectional design where participants underwent a continuous computed tomography (CT) scan (Siemens Somatom Sensation 64 Scanner), from the 2^nd^ lumbar vertebra (L2) to the patella while lying in supine position. From the whole scan, three regions of interest were selected for analysis, abdominal, hip and mid-thigh regions, specifically at the level of the 3^rd^ lumbar vertebrae, 3^rd^ sacral vertebrae and 50% of femur’s length respectively (Figure 1). The following muscles were selected: Psoas (PS), Gluteus Maximus (GMax), Gluteus Medius and Minimus (GMm), Vastus Lateralis (VL), Rectus Femoris (RF), Hamstrings (Ham) compartment and Adductor Magnus and Longus (Add). Muscle cross section area (CSA), Intramuscular adipose tissue (IMAT) content and muscle attenuation were determined with MIPAV (Medical Image Processing, Analysis and Visualization, v 7.0, NIH) analysis software by tracing the fascia around the selected muscles. Scanning and analysis procedures have been previously reported in detail [24], with CT data expressed as cross-sectional area of tissue (cm2), and using Hounsfield units (HU) for muscle area between 30 to 80 HU, fat as −190 to −30 HU, and low density lean tissue as 0–29 HU [24]. In addition, the IMAT content was normalized to the respective muscle’s size $\left(\mathrm{normalized}\;\mathrm{IMAT}=100*\frac{\mathrm{IMAT}}{\mathrm{CSA}}\right)$ and expressed as a percentage of the muscle’s CSA (cm^2^) [29].

Height (cm) and weight (kg) were measured to calculate body mass index (*BMI*, *kg*/*m*^*2*^). Fat mass and lean tissue mass (fat-free mass = lean + bone) were determined by Dual-energy X-ray Absorptiometry (Prodigy, LUNAR Radiation Corp., Madison, WI). A trained radiology technician performed all DXA scans. Our past referenced CV’s for total body percent fat, fat tissue mass, lean tissue mass, and BMC are 1.4%, 1.4%, 0.7%, and 0.4%, respectively [30].

Isokinetic strength testing (Biodex System 4, Biodex Medical Systems, Shirley, NY) performed by blinded trained research staff involved performing bilateral concentric contractions at 60^o/s^ for knee flexion-extension (seated), hip flexion-extension (supported standing), and hip abduction-adduction (supported standing). An external stabilization frame was used to minimize extraneous body movements while standing similar to previous study [7]. Knee flexion-extension was performed from 90° to 5° of knee flexion, hip flexion-extension was performed from 0° to 45° of hip flexion and hip abduction-adduction was performed from 0° to 30° of hip abduction. Due to a change in isokinetic measurement system used during the study and to procedural errors on some of the tests, the number of participants used in the analysis of the isokinetic performance variables was 12 non-fallers and 10 fallers. All peak torque data were normalized to height and weight of each subject using the following equation:

$$\mathrm{Normalized}\;\mathrm{Torque}=\frac{\mathrm{Peak}\;\mathrm{Torque}}{\mathrm{Height}*\mathrm{Weight}}$$

### Statistical analyses

Statistical analyses were performed using SPSS (v.17) statistical software. Considering the muscle composition measures did not have a normal distribution (Shapiro-Wilk test, *p* < *0.05*), comparisons between non-fallers and fallers groups on muscle attenuation, IMAT and CSA were performed by a Mann–Whitney U test. Between-muscle comparisons on each individual group were performed by a Kruskal-Wallis one-way analysis of variance, followed by a series of Mann–Whitney U tests to test for pairwise comparisons where Bonferroni correction for multiple comparisons was applied. Muscle performance measures were normally distributed (Shapiro-Wilk test, *p* > *0.05*), thus between-group comparisons of peak joint torque were performed by independent samples t-test. Associations between joint torque and muscle composition data were performed using Pearson’s correlation. Significance was set at *p* < 0.05.

## Results

Age, height, weight and BMI were not significantly different between groups. Approximately two thirds of the faller group experienced a single fall in the previous 12 months while the remainder of the group had two or more falls (Table 1). In accordance with the exclusion criteria, the existing co-morbidities are presented in Table 1. Due to the different gender ratio in the groups, gender was used as a covariate to assess any potential gender differences on the different groups. The results from this initial analysis of covariance showed that both genders were similar in every muscle and fall classification group for percent IMAT and muscle attenuation, with only the exceptions of Psoas and Vastus Lateralis muscles in the non-faller group for normalized IMAT (see Additional file 1, Table A1 for further details). Considering that, in general, gender appeared not to impact the present results, the entire data set was used in the analyses and described below.

### Between-muscle comparisons

Multiple significant differences were found between muscle groups (see Additional file 2, Table A2 for the complete analysis). Overall, Gluteus Maximus muscle attenuation was lower than all of the other muscles (*p* < 0.0024), whereas Rectus Femoris had the highest muscle attenuation (*p* < 0.0024). In the non-faller group, Gluteus Medius/Minimus and Adductors had significantly higher muscle attenuation than Gluteus Maximus but lower values than all other muscles (*p* < 0.0024). In the faller group, Gluteus Medius/Minimus, Vastus Lateralis and Adductors had similar muscle attenuation (*p* > 0.0024), which was greater than Gluteus Maximus but less than all of the other muscles (*p* < 0.0024).The percentage of IMAT infiltration was highest for Gluteus Maximus (*p* < 0.0024) and lowest for Rectus Femoris (*p* < 0.0024) in both groups. In the non-faller group, the relative adipose infiltration was similar between Gluteus Medius/Minimus and Gluteus Maximus (*p* > 0.0024). In the faller group, Gluteus Medius/Minimus and Psoas muscles had similar relative adipose tissue infiltration (*p* > 0.0024) that was significantly less than that for Gluteus Maximus, but more than for the other muscles (*p* < 0.0024).

### Between-group comparisons

**Figure 2:**
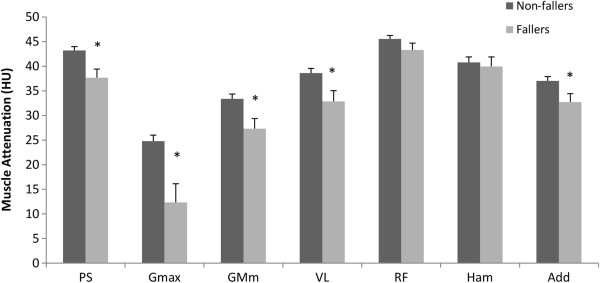
**Comparison between non**-**faller and faller groups for muscle attenuation (*HU*).** Data expressed as Mean ± SEM. * indicates significant difference (*p* < 0.05).

**Figure 3:**
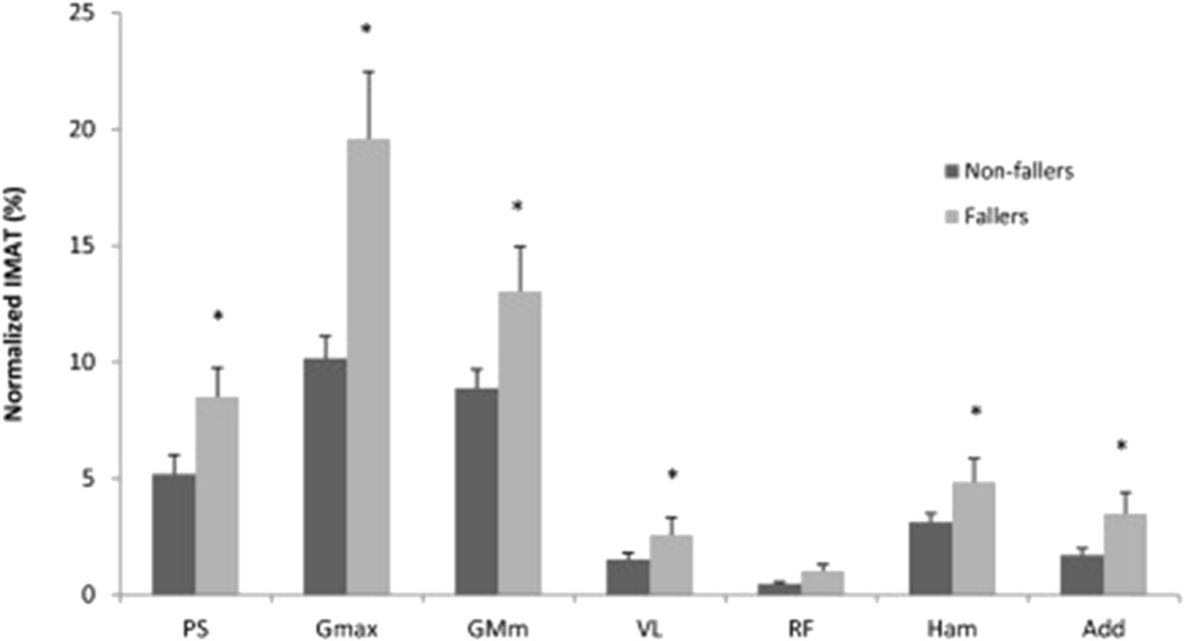
**Comparison between non**-**faller and faller groups for normalized intramuscular adipose tissue (IMAT) (%).** Data expressed as Mean ± SEM. *indicates significant difference (*p* < 0.05).

Muscle CSA was similar between groups for all of the muscles (*p* > 0.05). However, the non-faller group had higher muscle attenuation values for most muscles compared with the faller group (*p* < 0.05, Figure 2). Likewise, percent IMAT in the Psoas, Rectus Femoris, Hamstrings, Adductors, Gluteus Maximus and Gluteus Medius/Minimus was higher in the faller group (*p* < 0.05, Figure 3). Normalized peak joint torque differed between the groups only for hip abduction, which was greater for the non-fallers (*p* < 0.05, Table 2).

**Table 2:** **Comparison between non**-**faller and faller groups on normalized peak isokinetic torque** (**N.Kg **^−**1**^)

	**Non**-**fallers**	**Fallers**	***p***
	**( *n *** **= *12 *)**	**( *n *** **= *10 *)**	
Knee Extension	0.71 ± 0.03	0.61 ± 0.03	*0.06*
Knee Flexion	0.31 ± 0.01	0.32 ± 0.02	*0.64*
Hip Extension	0.37 ± 0.03	0.36 ± 0.03	*0.73*
Hip Flexion	0.59 ± 0.02	0.54 ± 0.03	*0.12*
Hip Abduction	0.53 ± 0.02	0.41 ± 0.03	* *0.01*
Hip Adduction	0.22 ± 0.02	0.22 ± 0.02	*0.90*

Data is expressed as Mean ± SEM. *indicates significant difference (*p* < 0.05).

### Associations between joint torque and muscle composition

Hip abduction torque was inversely associated with Gluteus Medius/Minimus percent IMAT infiltration (*r* = −0.49, *p* < 0.01) and positively associated with muscle attenuation (*r* = 0.53, *p* < 0.01). Hip extension torque was inversely associated with Gluteus Maximus percent IMAT infiltration (*r* = −0.39, *p* < 0.05). There was a trend for an association between hip extension torque and Gluteus Maximus muscle attenuation (*r* = 0.31, *p* = 0.056). Similarly, hip flexion torque was inversely associated with Psoas percent IMAT infiltration (*r* = −0.46, *p* < 0.01) and positively associated with muscle attenuation (*r* = 0.34, *p* < 0.05). Knee flexion torque was associated with Hamstrings muscle attenuation (*r* = 0.41, *p* < 0.01), but not with IMAT infiltration. Additionally, the normalized knee extension torque was not associated with any of the muscle composition measures for the Vastus Lateralis and Rectus Femoris. A similar lack of associations was observed between hip flexion torque and Rectus Femoris, as well as between hip adduction torque and Adductors.

## Discussion

This was among the first studies to investigate whether age-related changes in lower limb muscle composition and performance differentiated between faller and non-faller cohorts of older adults who were reportedly not residing in community care or other health care settings, and have not scored zero in any item of the Instrumental Activities of the Daily Living (IADL) impairment screening tool. Twenty-six percent of the participants reported falling one or more times during the yearlong monitoring period prior to testing. This incidence of falls generally resembled past studies [3,7,8,31].

### Between-muscle comparison

As hypothesized, muscle composition changes differed between lower limb muscles. Muscle attenuation analysis revealed several differences between the selected muscles across the two groups, where Rectus Femoris had the highest values and Gluteus Maximus and Gluteus Medius/Minimus showed the lowest values. These data suggested that the gluteal muscles may be particularly susceptible to composition changes with advancing age as evidenced by lower muscle density and reduced force generating capacity compared with other muscles of the lower limb [20].

Considering that muscle attenuation is inherently related to muscular adipose content [32], our findings for the adipose tissue infiltration were not surprising. In both groups, when adiposity was expressed relative to the specific muscle’s cross-sectional area, we observed that Gluteus Maximus and Gluteus Medius/Minimus showed the highest relative infiltration of adipose tissue. Although previous reports have demonstrated age-related differences in composition between different muscles [16,21,27], we found the most prominent composition changes for the proximal muscles of the hip. This novel finding has clinical relevance as it indicates that disproportionate changes may occur involving the proximal hip musculature. These changes may not only affect overall muscle function, but may especially impact the ability to stabilize standing balance in the frontal plane. In this connection, osteoarthritis is associated with hip muscle dysfunction and impaired balance [33,34]. Although only a small percentage of our study participants reported some type of mild osteoarthritis that did not affect daily living activities, it is possible that even asymptomatic osteoarthritic changes in the hip joints may be contributing to the observed muscle composition changes.

### Between-group comparison

Muscle CSA was not different between the groups for any of the muscles, and therefore does not likely confound the interpretation of the results. Nonetheless, even though the classical measurement of sarcopenia (muscle size) was not different between the groups, other muscle composition changes, such as skeletal muscle density and adipose infiltration, may be different between individuals and potentially impact performance and functional activities.

To our knowledge, this study has identified previously unreported differences in muscle composition between older fallers and non-fallers. Fallers had lower muscle attenuation in most muscle groups, with Gluteus Maximus and Gluteus Medius/Minimus showing the greatest reductions in muscle density. In addition, normalized IMAT was higher in the faller group for most of the muscle groups but especially for Gluteus Maximus and Gluteus Medius/Minimus. In particular, IMAT infiltration in Gluteus Maximus of the fallers was double that of the non-fallers. These findings differentiating between fallers and non-fallers are especially noteworthy because they were not just attributable to increased BMI as these were non-obese older adults. While several muscle composition measures identified differences between the groups, these differences were only reflected in impaired performance for the hip abductor muscles, where fallers were weaker than non-fallers.

### Associations between joint torque and muscle composition

Because of the inherent inverse relationship between IMAT infiltration and muscle attenuation, it was expected that joint torque and muscle attenuation would be positively associated, while torque and adipose infiltration would be negatively associated. These results indicated that the muscle composition changes were not only greater among fallers but were also associated with their poorer muscle performance.

When considered collectively, the findings indicated that changes in lower limb muscle composition in general, and of the gluteal muscles in particular, distinguished between older fallers and non-fallers. It is widely recognized that sarcopenic changes are related to loss of muscle mass, reduced muscle strength, physical frailty, and increased risk of fracture [19,26]. Such changes have also been associated with loss of mobility [25,27,35,36] and limitations in balance function [37]. For example, increased muscle fat infiltration has been shown to be related to reduced knee extensor muscle strength, as well as future mobility limitations among well-functioning older individuals [25,36]. Similarly, IMAT accumulation in the thigh has been identified as a predictor of 6 minute walk distance, stair ascent/descent, and Timed Up and Go time [35]. Among older adults with a history of back pain, increased trunk muscle fat infiltration predicted diminished functional capacity especially for balance outcomes [38]. Hence, the present findings have implications for mobility disability related to falls among community dwelling older individuals.

With respect to balance function, protective stepping is normally a primary strategy for recovering balance in the everyday environment that becomes more prevalent and problematic for those at greater risk for falls, especially in the mediolateral direction [8,10,11]. An age-associated reduction in the maximum hip abductor-adductor muscle torque generating capacity has been linked with impaired protective stepping in the lateral direction and prospective risk for falls [7,14]. Although the non-fallers in this study demonstrated greater torque production than fallers only for hip abduction, we generally found significant associations between peak joint torques and muscle composition. The strongest associations were found for Gluteus Medius/Minimus muscles that contribute to hip abduction torque and are important for lateral balance stability. We propose that the present results may be indicative of an underlying mechanism contributing to functional deficits in frontal plane balance stability experienced by older individuals at greater risk for falls [37,39]. The substantial composition changes for Gluteus Maximus among the faller group also has relevance to lateral stability during gait where older adults have demonstrated an increased reliance on Gluteus Maximus (hip and back extensor muscle) contributions to controlling mediolateral balance when compared to younger adults [40]. Additionally, as a primary hip extensor muscle, Gluteus Maximus IMAT infiltration could contribute to impaired recovery from a trip or slip while walking via forward or backward protective stepping [37,39,41]. From a rehabilitation perspective, previous studies have shown that muscle composition can be improved with exercise training and nutrition in older individuals [23,42-46]. Therefore, the present results may help to guide exercise interventions that target proximal hip muscle composition and performance to enhance balance and mobility, two important factors in fall prevention.

Among the limitations of this study is the extent to which the results may be more generally applicable to older individuals beyond community dwelling older adults who are relatively healthy and functionally independent. Thus, frail older people with other morbidities affecting functional capacity may present different muscle composition profiles than those identified here. Additionally, although we found significant associations between muscle composition and muscular performance, the cross-sectional design of the study has a limited potential for inferring causality. A small and similar percentage of individuals in both groups reported experiencing some type of osteoarthritis, which has been associated with reduced muscle composition and quality [16,47]. Although unlikely, it is possible that it may be impacting the outcome measurements. Furthermore, the statistical power for the strength measurements may have also been affected due to the smaller sample size used in this particular test. Another limitation is that we did not account for possible differences in participants’ physical activity levels. However, the impact of this limitation may have been minimized as none of the subjects were participating in a regular vigorous exercise program. The smaller sample size in the faller group was a further limitation of the study. However, the incidence of falls involving 26% of our subjects generally resembles prior studies [3,7,8,31]. It is also acknowledged that the retrospective self-report for falls may have underestimated their true incidence compared with a calendar method [48]. Nevertheless, self-report remains as an important source of information about falls occurring in the community and our present data may bolster the notion that individuals self-reporting falls are physiologically distinct from those who do not.

## Conclusions

In summary, the findings indicated that age-related changes in muscle composition are not equivalent throughout the lower limb musculature. While fallers were differentiated from non-fallers by greater IMAT infiltration and greater reduction in muscle attenuation of several muscles, the most affected muscles were Gluteus Maximus, Medius and Minimus. These regionally disparate changes in muscle composition may influence directional changes in lateral balance function, and possibly, successfully recovering from a trip while walking or backward falls.

## Competing interests

The authors have no conflicts of interest related to the content of this manuscript.

## Acknowledgments

The authors acknowledge the Claude D. Pepper Older Americans Independence Center, University of Maryland School of Medicine, Baltimore, MD, USA, and the assistance of D. Yungher, J. Morgia and K. Riddle. The helpful comments of Dr. Odessa Addison on a draft manuscript are gratefully acknowledged. This work was supported by the National Institute on Aging at the National Institutes of Health (R01AG029510, P30AG028747), Claude D Pepper - Older Americans Independence Center Grant (OAIC) NIH/NIA P30 AG028747, University of Maryland Advanced Neuromotor Rehabilitation Research Training (UMANRRT) Program, supported by the National Institute of Disability and Rehabilitation Research post-doctoral training grant (H133P100014) and a VA Research Career Scientist Award to Alice S. Ryan.

## Authors’ contributions

MI carried out data collection and analysis, and drafted the manuscript. AR helped to draft the manuscript and with CT analysis or data analysis. WB and BB participated in the design of the study. MP participated in the design and coordination. MR conceived the study and helped to draft the manuscript. All authors read and approved the final manuscript.

## Pre-publication history

The pre-publication history for this paper can be accessed here:

http://www.biomedcentral.com/1471-2318/14/37/prepub

## Supplementary Material

Additional file 1: Table A1 Between-gender comparisons in the non-faller and faller groups. Click here for file

Additional file 2: Table A2 Between-muscle comparisons in the non-faller and faller groups. Click here for file
