# Supplementary material for: Gluteal muscle composition differentiates fallers from non-fallers in community dwelling older adults
Source: BMC Geriatr. 2014 Mar 25;14:37. doi: 10.1186/1471-2318-14-37 (PMC4101852; doi:10.1186/1471-2318-14-37)
Supplement: Additional file 1: Table A1 — Between-gender comparisons in the non-faller and faller groups. [file 1471-2318-14-37-S1.pdf]

Table A1. Between-gender comparisons in the non-faller and faller groups, on muscle attenuation (*HU*) and normalized IMAT (%).

		Non-fallers			Fallers	
		Men (*n*=21)	Women (*n*=22)		Men (*n*=5)	Women (*n*=10)
Attenuation (HU)	PS	43.55 ± 1.19	42.58 ± 0.99	PS	39.05 ± 2.66	36.05 ± 2.42
	GMax	26.66 ± 1.63	23.41 ± 1.64	GMax	13.13 ± 6.37	11.47 ± 4.60
	GMm	32.12 ± 1.72	34.58 ± 1.03	GMm	31.45 ± 2.90	24.88 ± 2.62
	VL	39.40 ± 1.48	37.74 ± 1.12	VL	33.04 ± 3.61	32.21 ± 2.70
	RF	46.66 ± 0.89	44.29 ± 0.99	RF	41.09 ± 2.42	44.00 ± 1.66
	Ham	41.10 ± 1.62	40.68 ± 1.42	Ham	39.07 ± 3.31	40.66 ± 2.34
	Add	37.13 ± 1.34	36.49 ± 1.09	Add	35.14 ± 3.29	30.97 ± 1.87
Normalized IMAT (%)	PS	7.52 ± 1.58	3.28 ± 0.43 *	PS	7.90 ± 2.28	9.07 ± 1.51
	GMax	10.13 ± 1.64	10.04 ± 1.00	GMax	18.55 ± 4.06	20.35 ± 3.71
	GMm	10.33 ± 1.52	7.43 ± 0.60	GMm	11.56 ± 2.47	13.70 ± 2.53
	VL	1.09 ± 0.34	1.85 ± 0.43 *	VL	2.13 ± 0.40	2.80 ± 1.08
	RF	0.31 ± 0.08	0.67 ± 0.14	RF	1.22 ± 0.61	0.90 ± 0.25
	Ham	3.44 ± 0.69	2.84 ± 0.34	Ham	3.71 ± 0.85	5.50 ± 1.37
	Add	1.78 ± 0.49	1.60 ± 0.30	Add	2.51 ± 0.83	4.23 ± 1.30

Data is expressed as Mean ± SEM. PS: psoas; GMax: gluteus maximus; GMm: gluteus medius/minimus; VL: vastus lateralis; RF: rectus femoris; Ham: hamstrings; Add: adductor magnus/longus. * indicates significant difference ($p < 0.05$).
