# Supplementary material for: Gluteal muscle composition differentiates fallers from non-fallers in community dwelling older adults
Source: BMC Geriatr. 2014 Mar 25;14:37. doi: 10.1186/1471-2318-14-37 (PMC4101852; doi:10.1186/1471-2318-14-37)
Supplement: Additional file 2: Table A2 — Between-muscle comparisons in the non-faller and faller groups. [file 1471-2318-14-37-S2.pdf]

Table A2. Between-muscle comparisons in the non-faller and faller groups, on muscle attenuation (*HU*) and normalized IMAT (*%*).

		Non-fallers							Fallers						
		RF	PS	VL	Add	Ham	Gmed	Gmax	RF	PS	VL	Add	Ham	Gmed	Gmax
Muscle Attenuation (HU)	RF			*	*	*	*	*			*	*		*	*
	PS			*	*		*	*						*	*
	VL	*	*				*	*	*						*
	Add	*	*					*	*						*
	Ham	*					*	*						*	*
	Gmed	*	*	*		*		*	*	*			*		*
	Gmax	*	*	*	*	*	*		*	*	*	*	*	*	
Normalized IMAT (%)	RF		*	*	*	*	*	*		*		*	*	*	*
	PS	*		*	*		*	*	*		*	*			*
	VL	*	*			*	*	*		*			*	*	*
	Add	*	*			*	*	*	*	*				*	*
	Ham	*		*	*		*	*	*		*			*	*
	Gmed	*	*	*	*	*			*		*	*	*		
	Gmax	*	*	*	*	*			*	*	*	*	*		

PS: psoas; GMax: gluteus maximus; GMm: gluteus medius/minimus; VL: vastus lateralis; RF: rectus femoris; Ham: hamstrings; Add: adductor magnus/longus.
* indicates significant difference ($p < 0.0024$).
